# Corrigendum: Coping with brief periods of food restriction: mindfulness matters

**DOI:** 10.3389/fnagi.2014.00345

**Published:** 2015-01-13

**Authors:** Brielle M. Paolini, Jonathan H. Burdette, Paul J. Laurienti, Ashley Morgan, Donald A. Williamson, W. Jack Rejeski

**Affiliations:** ^1^Laboratory for Complex Brain Networks, Radiology Department, Wake Forest School of MedicineWinston Salem, NC, USA; ^2^Translational Science Center, Wake Forest UniversityWinston Salem, NC, USA; ^3^Pennington Biomedical Research Center, Louisiana State UniversityBaton Rouge, LA, USA; ^4^Departments of Health and Exercise Science and Geriatric Medicine, Wake Forest UniversityWinston Salem, NC, USA

**Keywords:** networks, mindfulness, food cues, obesity, aging, craving, self-efficacy

There is a minor error in the results section of the aforementioned publication. The current text on page 11, lines 340–344 reads:

Conversely, as shown in Figure 3, the low mindful group had the greatest global efficiency in the auditory and insular cortices. An ROI analysis revealed that global efficiency in the insula/auditory cortex was significantly greater in the Low MAAS group compared to the High MAAS group for BOOST® (*P* = 0.01) and NO BOOST® (*p* = 0.02) conditions.

It should read as follows:

Conversely, as shown in Figure 3, the low mindful group had greater clustering of globally efficient nodes in the auditory and insular cortices. However, an ROI analysis revealed that the average global efficiency in the insula/auditory cortex was significantly greater in the High MAAS group compared to the Low MAAS group for both the BOOST® (*p* = 0.01) and NO BOOST® (*p* = 0.02) conditions. Thus, while the high global efficiency nodes were more densely clustered in the insula/auditory cortex for the Low MAAS group (**Figure 3**), the average global efficiency of those nodes was on average lower than the High MAAS group (**Table 2**).

## Conflict of interest statement

The authors declare that the research was conducted in the absence of any commercial or financial relationships that could be construed as a potential conflict of interest.

